# Gene Signature-Based Prognostic Model for Acute Myeloid Leukemia: The Role of *BATF*, *EGR1*, *PD-1*, *PD-L1*, and *TIM-3*

**DOI:** 10.7150/ijms.108527

**Published:** 2025-03-19

**Authors:** Yupei Zhang, Zhixi Chen, Jiamian Zheng, Shaohua Chen, Liye Zhong, Jie Chen, Cunte Chen, Songnan Sui, Yangqiu Li

**Affiliations:** 1 Key Laboratory for Regenerative Medicine of Ministry of Education, Institute of Hematology, School of Medicine, Jinan University, Guangzhou 510632, China.; 2 Department of Hematology, First Affiliated Hospital, Jinan University, Guangzhou 510632, China.; 3 Department of Hematology, Guangzhou First People's Hospital, South China University of Technology, Guangzhou 510180, China.; 4 Central People's Hospital of Zhanjiang, Zhanjiang, China.; 5 Zhanjiang Key Laboratory of Leukemia Pathogenesis and Targeted Therapy Research, Zhanjiang, China.

**Keywords:** *BATF*, *EGR1*, allogenic hematopoietic stem cell transplantation, acute myeloid leukemia, T cell exhaustion

## Abstract

**Background:** Acute myeloid leukemia (AML) is a malignancy of hematopoietic stem and progenitor cells, with T cell exhaustion linked to poor outcomes. Our previous research has shown that basic leucine zipper ATF-like transcription factor (*BATF*) and early growth response 1 (*EGR1*) play a role in chimeric antigen receptor T (CAR-T) cell exhaustion during AML tumor elimination. However, the roles of *BATF* and *EGR1* and their association with immune checkpoint genes in AML prognosis remain underexplored.

**Methods:** Bone marrow (BM) samples from 92 newly diagnosed AML patients at our clinical center (JUN-dataset) were analyzed to detect the expression levels of *BATF*, *EGR1*, programmed cell death 1 (*PD-1*), programmed death-ligand 1 (*PD-L1*), T cell immunoglobulin and mucin domain-containing protein 3 (*TIM3*) together with conducting a prognostic assessment. Our findings were validated using RNA sequencing data from 155 AML patients from the TCGA database and 199 AML patients from the Beat-AML database.

**Results:** High *BATF* expression correlated with poor overall survival (OS) (*P* = 0.030), whereas high *EGR1* expression indicated a favorable prognosis (*P* = 0.040). Patients with high *BATF* and low* EGR1* expression had worst outcomes (*P* < 0.001). Among those receiving allogenic hematopoietic stem cell transplantation (allo-HSCT), high *BATF* expression was linked to shorter OS (*P* = 0.004). Moreover, a prognostic model incorporating *BATF*, *EGR1*, *PD-1*, *PD-L1*, and *TIM-3* calculated a risk score, with high-risk patients demonstrating significantly shorter OS than low-risk patients in both total AML patients and allo-HSCT recipients (*P* < 0.001). Similar results were found in both the TCGA and Beat-AML datasets.

**Conclusions:** We establish a prognostic model based on *BATF*, *EGR1*, *PD-1*, *PD-L1*, and *TIM-3* expression that effectively predicts survival outcomes for AML patients and allo-HSCT recipients. This model may provide valuable insights for prognosis assessment and treatment strategies.

## Introduction

Acute myeloid leukemia (AML) is a highly heterogeneous malignant disease characterized by abnormal proliferation and differentiation blockade of myeloid hematopoietic stem and progenitor cells 1. Despite ongoing improvements in treatment strategies over recent years, overall outcomes for AML patients have improved modestly with refractoriness and high relapse rates remaining major clinical challenges 2-6. The 5-year survival rate for AML patients remains below 35% with patients over 60 years of age having a 5-year survival rate of less than 20% 7-9. With the rapid development and widespread application of next-generation sequencing (NGS) technologies, molecular biology has become an essential foundation for diagnosis, treatment decision-making, and prognosis assessment. The inclusion of genes such as *TP53*, *FLT3*, and *NPM1* in risk evaluation systems has improved the accuracy of risk stratification 10-12. However, approximately 40-50% of patients in the current risk stratification system are classified as intermediate-risk, where significant variability in prognosis exists. Existing stratification criteria struggle to accurately predict treatment response and long-term prognosis 13-16. To optimize the current risk stratification system, more precise predictive models are needed. Therefore, it is worth exploring novel biomarkers to assess prognosis and identify new therapeutic targets.

Continuous stimulation by tumor antigens can alter the phenotype and function of T cells, ultimately driving them into an exhausted state. This state is characterized by the upregulation of inhibitory receptors, such as programmed cell death 1 (*PD-1*), programmed death-ligand 1 (*PD-L1*), cytotoxic T-lymphocyte-associated protein 4 (*CTLA-4*), and T cell immunoglobulin and mucin domain-containing protein 3 (*TIM3*), impaired cell proliferation and cytokine secretion, compromised immune memory function, and metabolic dysregulation. Together, these changes weaken the immune response against tumors and adversely affect patient prognosis 17, 18. Increasing data have shown that T cell exhaustion is a key factor contributing to poor prognosis in AML patients 19, 20. In our previous research, we observed that the expression levels of these inhibitory receptor genes are closely correlated with the prognosis of patients with hematologic malignancies 21, 22. However, the correlation between immune checkpoint genes and genes that regulate T cell function remains unknown.

Basic leucine zipper ATF-like transcription factor (*BATF*) is an important transcription factor that is involved in immune regulation, cancer initiation and progression, and inflammation, and it plays a key regulatory role in the exhaustion of effector T cells 23, 24. High *BATF* expression is thought to inhibit the cytotoxic function of chimeric antigen receptor T (CAR-T) cells, promoting their transition into an exhausted state and ultimately weakening their antitumor efficacy 25-27. While the transcription factor early growth response 1 (*EGR1*), plays an important role in T cell differentiation and activation, upregulation of *EGR1* can enhance T cell activity and immune responsiveness, suggesting that modulating this gene may be a potential approach for boosting T cell functionality and delaying exhaustion 28, 29. In our previous research on CAR-T cell-mediated elimination of AML tumor cells, we found that *BATF* downregulation and *EGR1* upregulation are closely associated with reduced CAR-T cell exhaustion and enhanced cell functionality 30.

Based on these findings, further investigation into the roles of key factors such as *BATF* and *EGR1* in the prognosis of AML patients, as well as their relationships with immune checkpoint molecules, may provide new insight into the molecular mechanisms underlying T cell exhaustion. In this study, we first analyzed bone marrow (BM) samples collected from 92 AML patients at our clinical center, examining the association between the *BATF* and *EGR1* expression levels and patient prognosis with a particular focus on outcomes for patients receiving allogenic hematopoietic stem cell transplantation (allo-HSCT). Additionally, we developed a predictive model that integrates *BATF*, *EGR1*, and immune checkpoint gene (*PD-1*, *PD-L1*, and *TIM-3*) expression to evaluate the combined impact of these factors on prognosis. We subsequently validated the generalizability of our findings using two publicly available datasets (TCGA and Beat-AML).

## Materials and methods

### BM samples

Bone marrow samples from 92 newly diagnosed AML patients were collected at our clinical center from January 1, 2013 to December 31, 2023, forming the JUN-dataset. Inclusion criteria: new diagnosis of AML based on the Chinese guidelines for the diagnosis and treatment of Acute Myeloid Leukemia 31. Corresponding clinical characteristics, including gender, age, treatment regimen, survival time, and survival status, are detailed in Table S1. Follow-up was completed on August 31, 2024. The OS time was defined as the time from the date of diagnosis to death or the last follow-up. This study was conducted in accordance with the Helsinki Declaration and approved by the Ethics Committee of the First Affiliated Hospital of Jinan University. All participants signed informed consent forms.

### Publicly available datasets

The TCGA dataset, which includes RNA-seq data and clinical information from 155 AML samples, was downloaded from the UCSC Xena website (https://xenabrowser.net/datapages/) 32, 33. The Beat-AML dataset, comprising RNA-seq data and clinical information from 199 AML samples, was obtained from the Beat-AML database (http://www.vizome.org/aml/) 34. The RNA-seq data were presented as log_2_(RPKM + 1), and the clinical information encompassed age, gender, survival time, survival status, white blood cell count, and treatment (Table S1).

### Quantitative real-time PCR (qRT-PCR)

Total RNA was extracted from mononuclear cells from BM samples using TRIzol reagent (Invitrogen, Carlsbad, CA, USA) according to the manufacturer's instructions. The RNA was reverse transcribed into complementary DNA (cDNA) using a reverse transcription kit (Promega Corporation, Madison, Wisconsin, USA) 35, 36. The relative expression levels of *BATF, EGR1, PD-1, PD-L1*, and *TIM-3* were detected by qRT-PCR using a qRT-PCR kit (TIANGEN, Beijing, China). The qRT-PCR reaction procedures were as follows: preincubation, 95°C for 10 min and amplification, 95°C for 10 sec and 60°C for 20 sec for a total of 40 cycles. *β*-actin was selected as an internal control 37. The qRT-PCR primer sequences are shown in Table S2. The results are expressed as 2^(-ΔCT).

### Statistical analysis

Statistical analyses were performed using SPSS (version 25.0, IBM) and R (version 4.4.1, https://www.r-project.org/). The high or low level of gene expression data was determined by the optimal cutoff point, which is determined by the X-tile software (version 3.6.1) 38, 39. Differences in Kaplan-Meier curves were compared by utilizing the log-rank test via the R package "survival". Univariate and multivariate Cox regression analyses were performed using the R packages " survminer" and "survival". The area under the curve (AUC) in receiver operating characteristic (ROC) curves was determined by the R package "pROC". A p-value < 0.05 was considered statistically significant.

## Results

### High *BATF* or low *EGR1* expression is associated with poor OS in AML patients

**Figure 1:**
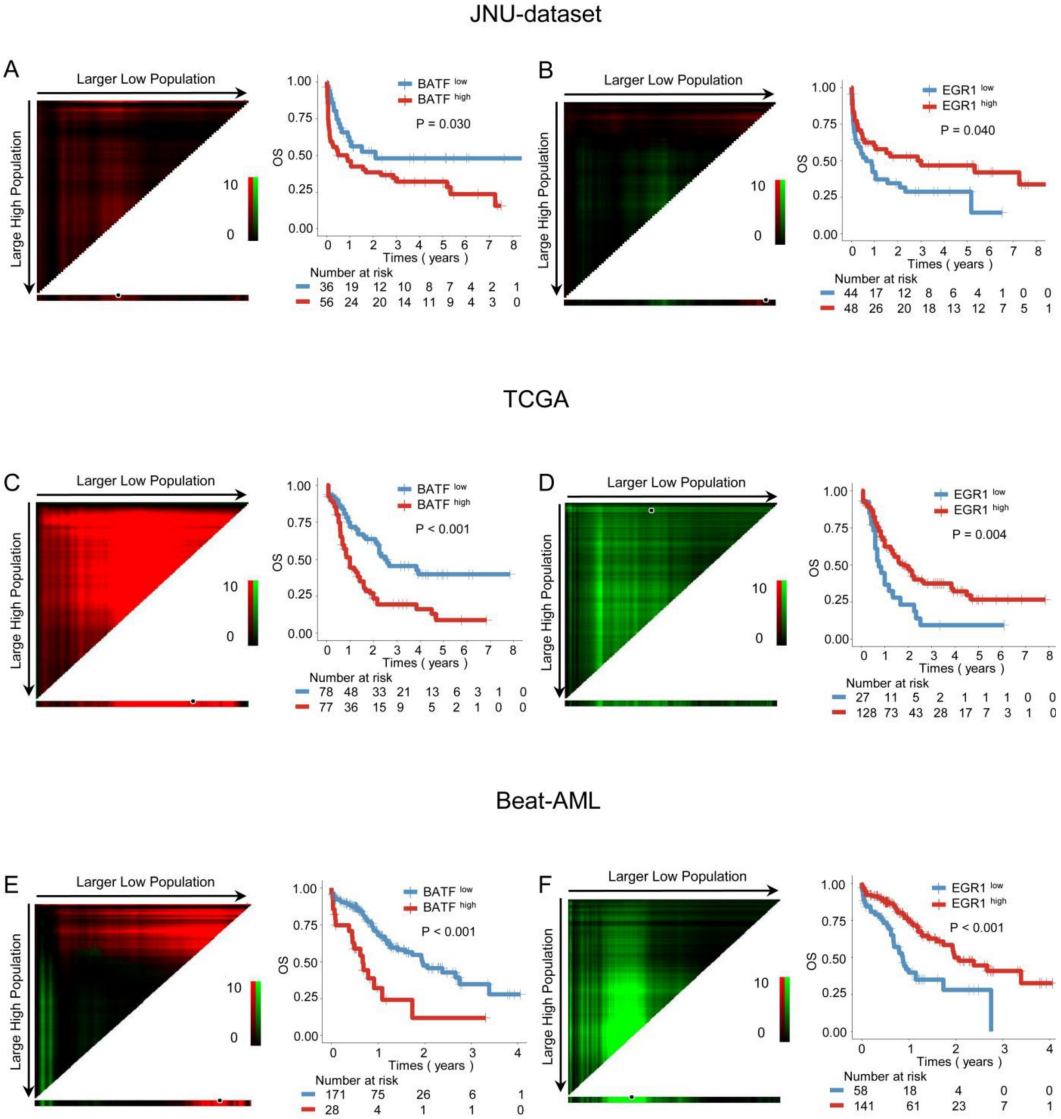
** Prognostic analysis of *BATF* and *EGR1* expression in AML patients.** A-B: Correlation between the expression levels of *BATF* or *EGR1* and overall survival (OS) in the JUN-dataset. (left panel) The optimal cut-point for *BATF* or *EGR1* was determined by X-tile software (version 3.6.1), which was shown as the highest pixel. (right panel) Kaplan-Meier curve was plotted according to the optimal cut-point. Blue and red indicate low and high expression of *BATF* or *EGR1*, which were plotted in Kaplan-Meier curves (top) with the number at risk AML patients (bottom). C-F: Relationship between the expression level of *BATF* or *EGR1* and OS in the TCGA (C, D) and Beat-AML (E, F) datasets.

Our previous research found that a BRD4 inhibitor could reverse the exhaustion of CAR-T cells in killing AML cells by downregulating *BATF* and upregulating *EGR1*. Therefore, we aim to further investigate the impact of the *BATF* and *EGR1* expression levels on prognosis for AML patients. Kaplan-Meier analysis demonstrated that high *BATF* expression was associated with poorer OS (3-year OS: 37.50% vs. 52.78%, *P* = 0.030) (Figure 1A), while high *EGR1* expression was also linked to longer OS (3-year OS: 52.08% vs. 34.09%, *P* = 0.040) (Figure 1B). Furthermore, when age, gender, white blood cell counts at diagnosis, treatment choice, *BATF* expression, and *EGR1* expression were included in univariate and multivariate Cox regression for survival analysis, the results indicated that *BATF* and *EGR1* are independent prognostic predictors of OS (HR = 2.656, 95% CI: 1.460-4.832, *P* = 0.001; HR = 2.092, 95% CI: 1.198-3.655, *P* = 0.009) (Table S3).

The above results were further verified in AML patient cohorts from database. In the TCGA cohort, AML patients with higher *BATF* expression had poorer prognosis (3-year OS: 27.27% vs. 56.41%, *P* < 0.001) (Figure 1C), Conversely, higher *EGR1* expression was linked to better prognosis (3-year OS: 46.88% vs. 18.52%, *P* = 0.004) in AML patients (Figure 1D). Similarly, in the Beat-AML cohort, high *BATF* expression was also correlated with poor outcome (3-year OS: 35.71% vs. 63.16%, *P* < 0.001) (Figure 1E), while high expression of *EGR1* was associated with good outcome (3-year OS: 66.67% vs. 39.66%, *P* < 0.001) (Figure 1F). Next, we conducted univariate and multivariate Cox regression analyses including age, sex, white blood cell counts at diagnosis, treatment choice, *BATF* expression, and *EGR1* expression in the TCGA and Beat-AML cohorts separately. The results demonstrated that *BATF* and *EGR1* are an also independent prognostic factors for OS (TCGA: HR = 2.287, 95% CI: 1.474-3.550, *P* < 0.001; HR = 2.227, 95% CI: 1.357-3.657, *P* = 0.002; Beat-AML: HR = 3.282, 95% CI: 1.855-5.810, *P* < 0.001; HR = 2.506, 95% CI: 1.585-3.961, *P* < 0.001) (Table S4).

### AML patients with high *BATF* expression together with low *EGR1* together have the poorest prognosis

**Figure 2:**
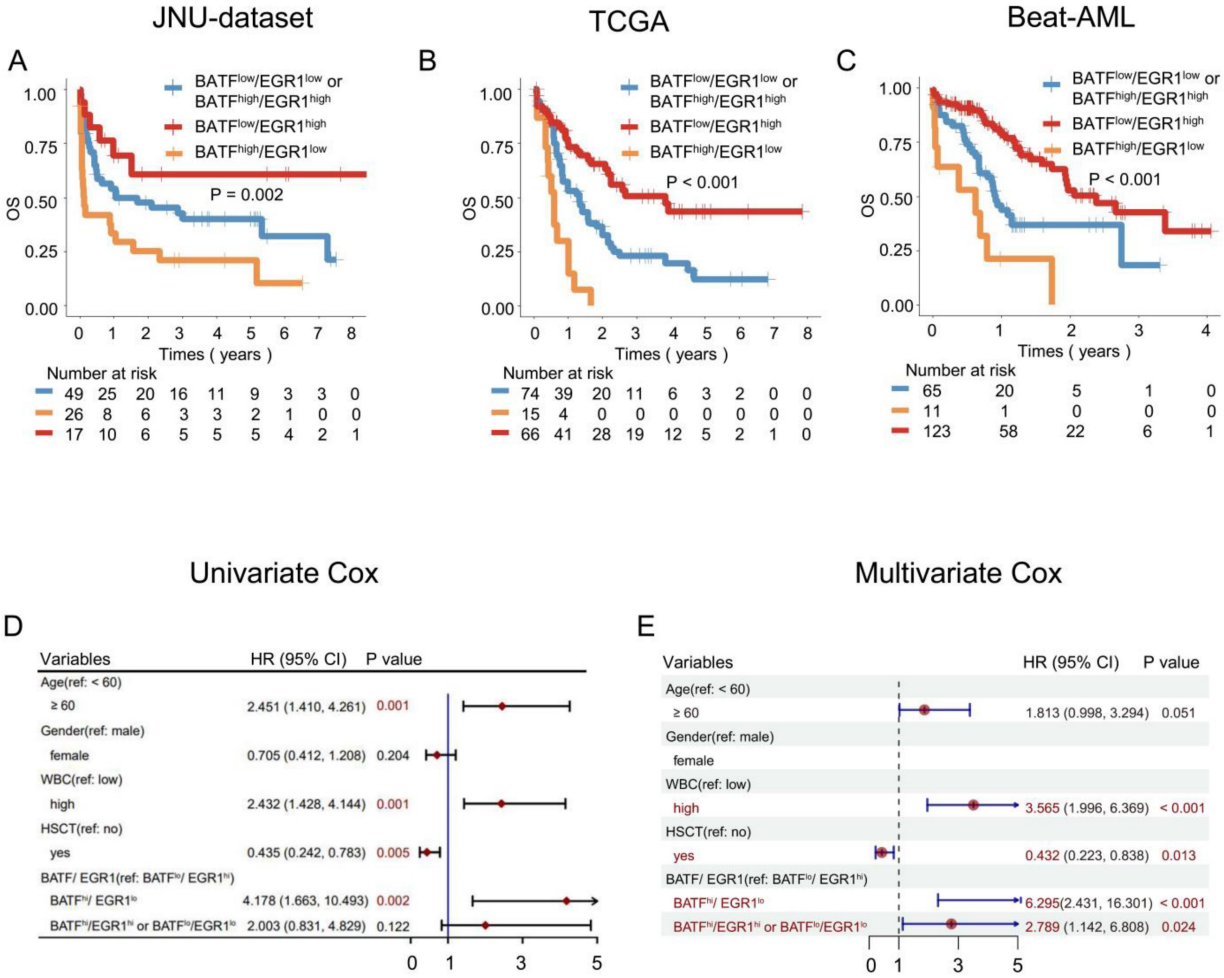
** Co-expression of the *BATF* and *EGR1* genes is associated with poor OS in AML patients.** A-C: Kaplan-Meier curves are shown for different *BATF* and *EGR1* combinations in the JUN-dataset (A). Kaplan-Meier curves are shown for different* BATF* and *EGR1* combinations in the TCGA (B) and Beat-AML (C) datasets. According to the optimal cut-off value, the *BATF* and *EGR1* genes were divided into low *BATF* expression and high *EGR1* expression (red line), high *BATF* expression and low *EGR1* expression (yellow line), low *BATF* expression and low *EGR1* expression or high *BATF* expression and high *EGR1* expression (blue line), which were plotted in Kaplan-Meier curves (top) with the number at risk AML patients (bottom). D-E: Univariate and multivariate Cox regression analysis of *BATF* and *EGR1* co-expression in JUN-dataset.

To investigate the role of *BATF* and *EGR1* co-expression in predicting OS for AML patients, we conducted a combined group analysis. In the JUN-dataset cohort, AML patients with high *BATF* expression and low *EGR1* expression had the poorest outcomes (3-year OS: 26.92% vs. 44.90% and 64.71%, *P* = 0.02) (Figure 2A). In the TCGA cohort, high *BATF* expression together with low *EGR1* expression was associated with the poorest OS (3-year OS: 6.67% vs. 32.43% and 60.60%, *P* < 0.001) (Figure 2B). Similarly, in the Beat-AML cohort, patients with high *BATF* expression and low *EGR1* expression had the shortest OS (3-year OS: 18.18% vs. 46.15% and 69.92%, *P* < 0.001) (Figure 2C). Through univariate and multivariate Cox regression analyses, high *BATF* and low* EGR1* expression was able to predict poor OS of AML patients in the JUN-dataset (HR = 6.295, 95% CI: 2.431-16.301, *P* < 0.001) (Figures 2D, E). Similar results were also found in the TCGA and Beat-AML cohorts (TCGA: HR = 5.365, 95% CI: 2.720-10.583, *P* < 0.001; Beat-AML: HR = 5.638, 95% CI: 2.607-12.191, *P* < 0.001) (Table S5).

### High *BATF* expression was associated with poor OS in AML patients receiving allo-HSCT

**Figure 3:**
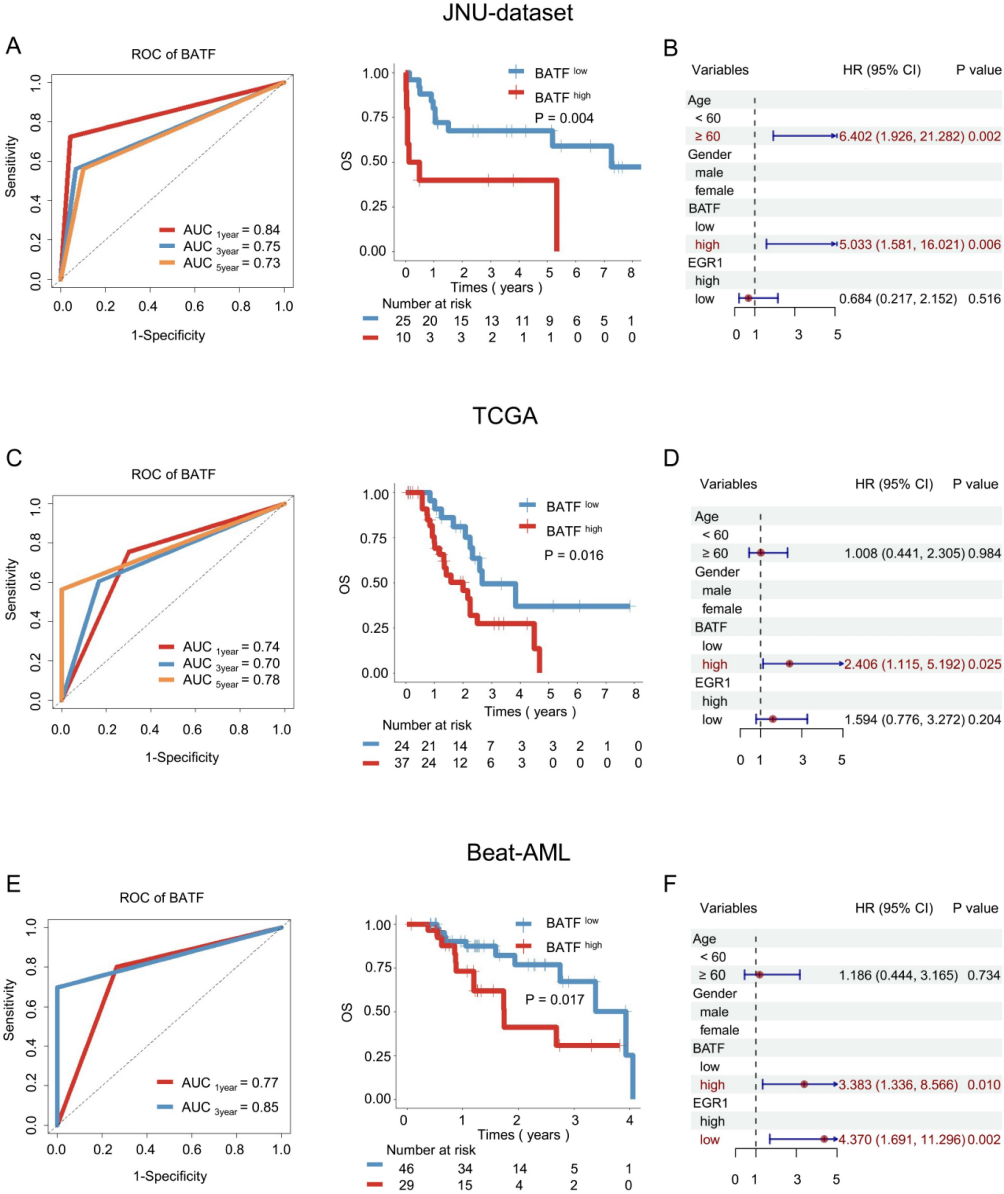
** BATF was associated with prognosis in AML patients undergoing allo-HSCT.** A: ROC curve (left panel) and overall survival analysis (right panel) of *BATF* in the JUN-dataset. According to the optimal cut-off value, the *BATF* genes was divided into low *BATF* expression (blue line) and high *BATF* expression (red line), which were plotted in Kaplan-Meier curves (top) with the number at risk AML patients (bottom). B: Multivariate Cox regression analysis of allo-HSCT patients in the JUN-dataset. C: ROC curve (left panel) and overall survival analysis (right panel) of *BATF* in the TCGA dataset. D: Multivariate Cox regression analysis of allo-HSCT patients in the TCGA dataset. E: ROC curve (left panel) and overall survival analysis (right panel) of *BATF* in the Beat-AML dataset. F: Multivariate Cox regression analysis of allo-HSCT patients in the Beat-AML dataset.

To further elucidate the potential of the *BATF* and *EGR1* gene expression levels as prognostic biomarkers for AML patients undergoing allo-HSCT, we conducted an in-depth analysis of a cohort of allo-HSCT recipients. In the JUN-dataset, ROC analysis indicated that *BATF* has strong prognostic predictive power (AUC_3year_ = 0.75) (Figure 3A). Kaplan-Meier analysis demonstrated that high *BATF* expression indicates poorer prognosis (3-year OS: 40.00% vs. 68.00%, *P* = 0.004) (Figure 3A). Furthermore, when age, gender, *BATF* expression, and *EGR1* expression were included in univariate and multivariate Cox regression for survival analysis, *BATF* emerged as an independent prognostic factor for OS (HR = 5.033, 95%CI: 1.581-16.021, *P* = 0.006) (Figure 3B and Figure S1B).

To validate the above findings, further analysis was conducted using external databases. In the TCGA cohort of AML patients who received allo-HSCT, *BATF* demonstrated good prognostic predictive ability (AUC_3year_ = 0.70) (Figure 3C), with high *BATF* expression associated with poorer survival (3-year OS: 27.27% vs. 56.41%, *P* = 0.016) (Figure 3C). Analysis of the Beat-AML cohort further confirmed the robust prognostic predictive power of *BATF* (AUC_3year_ = 0.85) (Figure 3E), where high *BATF* expression was linked to worse OS (3-year OS: 35.71% vs. 63.16%, *P* = 0.017) (Figure 3E). When age, sex, *BATF* expression, and *EGR1* expression were included in univariate and multivariate Cox regression for survival analysis, *BATF* was identified as an independent prognostic factor for OS (TCGA: HR = 2.406, 95% CI: 1.115-5.192, *P* = 0.025; Beat-AML: HR = 3.383, 95% CI: 1.336-8.566, *P* = 0.010) (Figures 3D, F and Figures S1D, F).

In the JUN-dataset, possibly due to the small sample size, Kaplan-Meier analysis indicated no statistically significant correlation between *EGR1* expression and survival outcome for transplant patients (*P* > 0.05) (Figure S1A). In the Beat-AML cohort, patients expressing high levels of *EGR1* demonstrated significantly improved survival outcome (*P* = 0.003) (Figure S1E). Analysis of the TCGA cohort showed that patients with elevated *EGR1* expression exhibited a notable trend toward favorable prognosis, although this trend did not reach statistical significance (Figure S1C).

### A model based on *BATF*, *EGR1*, and immune checkpoint gene expression can predict the prognosis of AML patients

It is known that upregulation of immune checkpoint factors is related to T cell exhaustion and may influence clinical outcome in AML. Therefore, we next sought to assess the relationship between the weighted combinations of *BATF*, *EGR1*, *PD-1*, *PDL-1*, *TIM-3*, and survival outcome. Multivariate Cox regression analysis was performed with these five genes.

**Figure 4:**
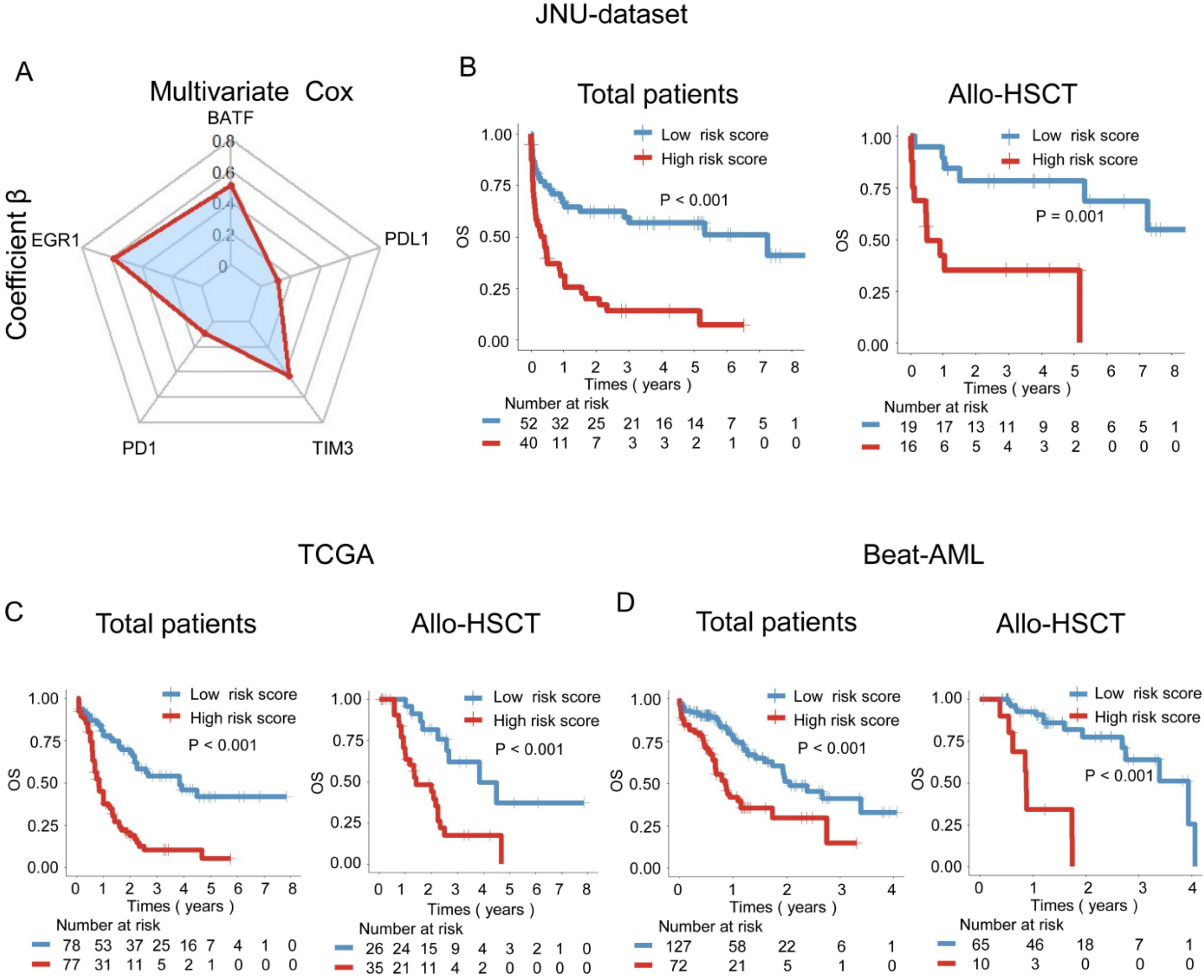
** A weighted combination of *BATF*, *EGR1*, and immune checkpoint gene expression levels is associated with AML patient prognosis.** A: Radar plot showing the contribution of the *BATF*, *EGR1*, *PD-1*, *PDL-1*, and *TIM-3* genes to OS in the JUN-dataset, which was determined by the coefficient β in the multivariate Cox regression model. Risk score = β1* (*BATF* expression) + β2* (*EGR1* expression) + β3* (*PD-1* expression) + β4* (*PDL-1* expression) + β5* (*TIM-3* expression). B: OS analysis of low-risk and high-risk score based on the combination of *BATF*, *EGR1*, *PD-1*, *PDL-1*, and *TIM-3* in the JUN-dataset for total patients (left panel) and those receiving allo-HSCT (right panel). C-D: Overall survival (OS) analysis of low-risk and high-risk scores calculated based on the prognostic model in the TCGA (C) and Beat-AML (D) datasets for total patients (left panel) and those receiving allo-HSCT (right panel). According to the optimal cut-off value, the risk score was divided into a low risk score (blue line) and a high risk score (red line), which were plotted in Kaplan-Meier curves (top) with the number at risk AML patients (bottom).

The following formula can be used to calculate the risk score of each patient according to the coefficients in our center's dataset: risk score = 0.60 × (*BATF* expression level) + 0.69 × (*EGR1* expression level) + 0.10 × (*PD-1* expression level) + 0.13 × (*PDL-1* expression level) + 0.50 × (*TIM-3* expression level) (Figure 4A). Importantly, patients with AML were divided into low-risk and high-risk score subgroups according to the optimal cut-point for the risk score. Patients with high-risk scores had significantly shorter OS than patients with a low-risk scores in the JUN-dataset (*P* < 0.001) (Figure 4B). Additionally, among patients who received allo-HSCT, those with high-risk scores had significantly poorer OS (*P* = 0.001) (Figure 4B). The results were further validated using the TCGA dataset, where high-risk patients also exhibited significantly poorer prognosis compared to low-risk patients (*P* < 0.001) (Figure 4C). Among patients who received allo-HSCT, those with high-risk scores had significantly shorter OS (*P* < 0.001) (Figure 4C). Similarly, in the Beat-AML cohort, high-risk patients demonstrated worse survival in both the overall patient population and the allo-HSCT subgroup (*P* < 0.001) (Figure 4D).

## Discussion

T cell exhaustion, which leads to T cell dysfunction, is a significant factor contributing to the poor prognosis of AML patients 40, 41. While the contribution of different exhaustion-related genes appears to be relatively different, defining the weight of single gene or the combined weight of such genes for T cell dysfunction as well as their association with prognosis for AML patients is worthwhile. Recent studies have identified *BATF* as a crucial regulator of T cell exhaustion, and the transcription factor *EGR1* plays an important role in T cell differentiation and activation 27, 29. Although there are a number of studies indicating that higher expression and co-expression of immune checkpoint genes such as *PD-1*, *PD-L1*, *CTLA-4*, and *TIM-3*, which induce T cell exhaustion, are associated with poor clinical outcomes for AML patients 42, 43 , it remains unclear whether alterations in T cell function genes alone or in cooperation with immune checkpoint factors contribute to T cell exhaustion and influence the clinical outcome of AML patients.

In this study, we first analyzed BM samples from 92 newly diagnosed AML patients at our center and observed high *BATF* expression and low *EGR1* expression. Our previous study, indicated that the upregulation of *BATF* and the downregulation of *EGR1* were closely associated with worsened CAR-T cell exhaustion and reduced cellular function. Additionally, several studies by other researchers have independently linked *BATF* or *EGR1* to T cell or CAR-T cell functionality 44, 45. Thus, we further investigated the association between alterations in the expression of both genes and clinical outcomes of AML patients. Indeed, we discovered that high *BATF* expression or low *EGR1* expression alone was closely associated with poor OS. Moreover, combined analysis revealed that patients with high *BATF* and low *EGR1* expression demonstrated significantly worse prognostic outcomes. This finding was also validated in two AML databases, suggesting that these genes may provide valuable insights for assessing AML prognosis.

It is recognized that one of the important influencing factors for overall survival is treatment strategy. Currently, allo-HSCT remains the only curative option for AML 46. T cell dysfunction is one of the reasons for AML relapse after allo-HSCT, and restoring and maintaining normal T cell function are important approaches for eliminating minimal residual disease (MRD) and preventing disease relapse 47, 48. Thus, alterations in T cell-regulating genes can serve as potential biomarkers evaluating the T cell immune state. Consequently, we conducted further analysis of an AML subgroup of patients who underwent allo-HSCT at our center. Significantly, *BATF* had strong prognostic predictive power with high expression of *BATF* associated with shorter survival for AML patients after allo-HSCT. As expected, high *EGR1* expression was related to better prognosis in the allo-HSCT group, while these results were revealed from the database analysis. In our center's cohort, significant association couldn't observe between EGR1 expression and overall survival in the allo-HSCT group, the reason may be attributed to the relatively small sample size within this subgroup, which compromises to accurately identify any underlying correlations. Further extent of the cohort and confirmation of the results are needed. Overall, these findings may at least provide a useful reference for pre-transplant prognostic risk assessment for AML patients undergoing allo-HSCT.

The upregulation of immune checkpoint factors is known to be associated with T cell exhaustion and may impact the prognostic survival of AML patients. Our previous studies have also indicated that the expression levels of immune checkpoint genes were correlated with the prognosis of patients with hematological malignancies 21. In this study, we further evaluated the relationship between the weighted combination of five genes (*BATF*, *EGR1*, *PD-1*, *PD-L1*, and *TIM-3*) and survival outcome. Using data from our center, we constructed a prognostic risk model, which indicated that high-risk score patients have significantly shorter survival times than low-risk score patients. Among patients receiving allo-HSCT, those with lower risk scores demonstrated improved survival outcomes. This finding was validated with two external databases. The impact of *BATF* and *EGR1* on AML prognosis may be related to T cell exhaustion mechanisms, providing valuable insight for developing novel therapeutic targets aimed at T cell exhaustion. Overall, these findings highlight that the *BATF* and *EGR1* expression levels are independent prognostic survival factors for AML patients. Elevated *BATF* expression is associated with poor prognosis in patients undergoing allo-HSCT. A prognostic model based on *BATF*, *EGR1*, and immune checkpoint gene expression effectively predicts prognosis for AML patients and those receiving allo-HSCT.

Nevertheless, the limitations of this study might include 1) as a single-center study conducted at our institution, it has a relatively small sample size and inherent selection bias and 2) further experiments are needed to elucidate the specific mechanisms by which *BATF* and *EGR1* regulate immune checkpoint genes in AML patients. 3) In this study, we tried to find the predicted factor on gene expression levels. As post-transcriptional, translational regulation and protein modification occur, protein and gene expression levels often differ. More research is needed to explore how the proteins translated from these genes affect prognosis in AML patients.

## Conclusions

We initially found that AML patients with high *BATF* expression or low *EGR1* expression have shorter survival times. Combining previous findings of alterations in immune checkpoint factors in AML, we for the first time established a prognostic model based on *BATF*, *EGR1*, *PD-1*, *PD-L1*, and *TIM-3* that effectively predicts survival outcomes for both AML patients and those receiving allo-HSCT. Our findings may offer valuable insights for prognosis assessment, monitoring disease progression, and informing treatment strategies.

## Supplementary Material

Supplementary figure and tables.

## Abbreviations

AML: Acute myeloid leukemia
BATF: basic leucine zipper ATF-like transcription factor
EGR1: early growth response 1
CAR-T: chimeric antigen receptor T
allo-HSCT: allogenic hematopoietic stem cell transplantation
PD-1: programmed cell death 1
PD-L1: programmed death-ligand 1
CTLA-4: cytotoxic T-lymphocyte-associated protein 4
TIM3: T cell immunoglobulin and mucin domain-containing protein 3
BM: bone marrow
