# Supplementary material for: Gene Signature-Based Prognostic Model for Acute Myeloid Leukemia: The Role of BATF, EGR1, PD-1, PD-L1, and TIM-3
Source: Int J Med Sci. 2025 Mar 19;22(8):1875–84. doi: 10.7150/ijms.108527 (PMC11983303; doi:10.7150/ijms.108527)
Supplement: Supplementary file 1 — Supplementary figure and tables. [file ijmsv22p1875s1.pdf]

## Supplementary figures and table

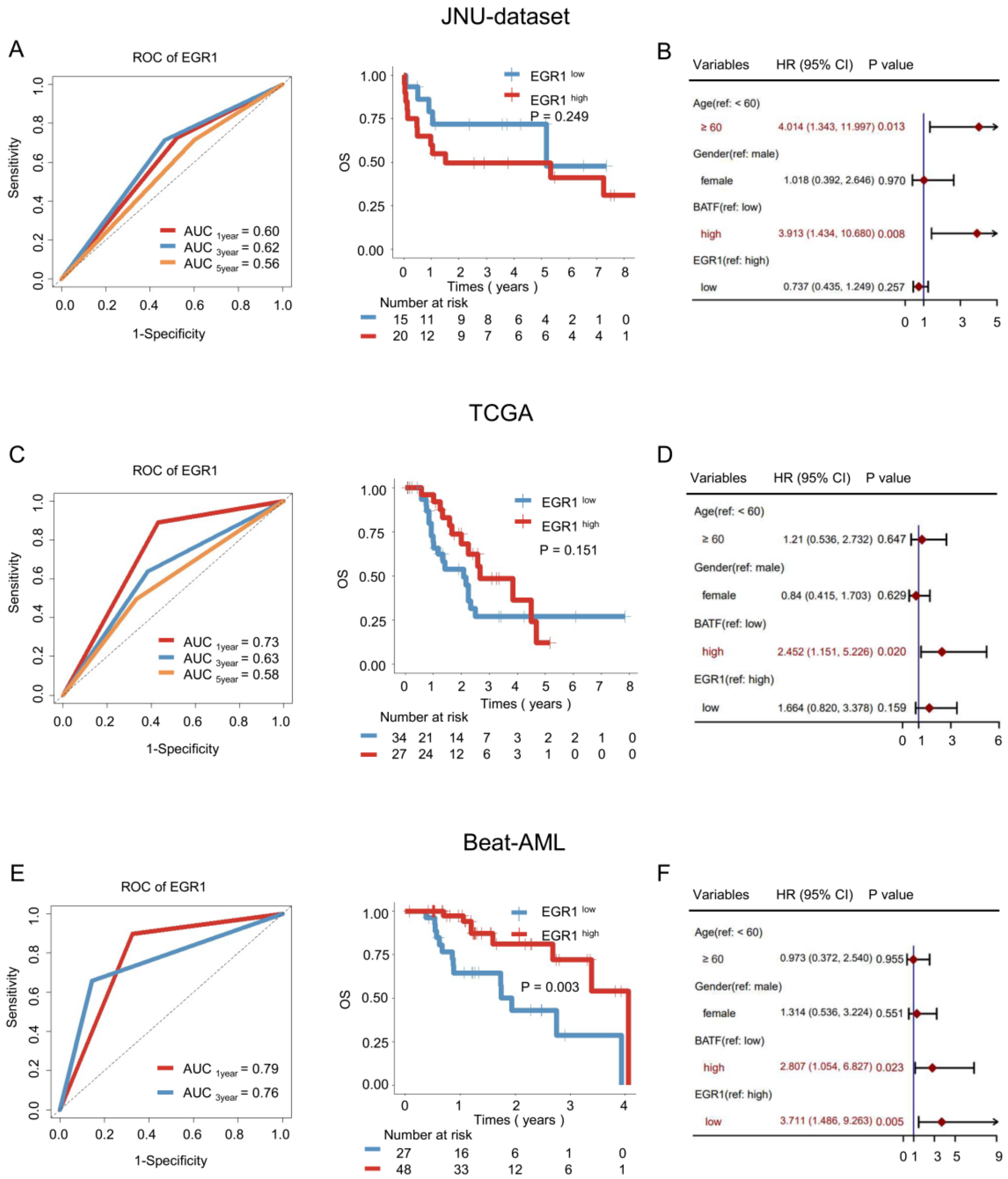


**Figure S1. Relationship between *EGR1* and prognosis for patients with AML patients undergoing allo-HSCT.** A: ROC curve (left panel) and overall survival analysis (right panel) of *EGR1* in the JUN-dataset. According to the optimal cut-off value, the *EGR1* genes was divided into low *EGR1* expression (blue line) and high *EGR1* expression (red line), which were plotted in Kaplan-Meier curves (top) with the number at risk AML patients (bottom). B: Univariate Cox regression analysis of allo-HSCT patients in the JUN-dataset.

C: ROC curve (left panel) and overall survival analysis (right panel) of *EGR1* in the TCGA dataset. D: Univariate Cox regression analysis of allo-HSCT patients in the TCGA dataset. E: ROC curve (left panel) and overall survival analysis (right panel) of *EGR1* in the Beat-AML dataset. F: Univariate Cox regression analysis of allo-HSCT patients in the Beat-AML dataset.

**Table S1. Clinical characteristics of AML patients.**

| Variables | JNU-dataset Patients (n = 92) | TCGA Patients (n = 155) | Beat-AML Patients (n = 199) |
|---|---|---|---|
| Age, mean ± SD, years | 50 ± 20 | 54±16 | 52±18 |
| Gender, n (%) | | | |
| male | 54 (58.7) | 82 | 95 |
| female | 38 (41.3) | 73 | 104 |
| WBC（×109/L）, mean ± SD | 45.0 ± 65.6 | 32.1 ± 38.0 | 17.1 ± 21.6 |
| Risk stratification, n (%) | | | |
| Low | 13 (14.1) | 32 (20.6) | 80 (40.2) |
| Intermediate | 33 (35.9) | 92 (59.4) | 49 (24.6) |
| High | 29 (31.5) | 31 (20.0) | 54 (27.1) |
| Unknow | 17 (18.5) | 0 | 16 (8.1) |
| Subtype, n (%) | | | |
| M0 | 1 (1.1) | 13 (8.4) | 4 (2.0) |
| M1 | 5 (5.4) | 35 (22.6) | 7 (3.5) |
| M2 | 21 (22.8) | 35 (22.6) | 6 (3.0) |
| M3 | 7 (7.6) | 15 (9.7) | 7 (3.5) |
| M4 | 13 (14.1) | 34 (21.9) | 20 (10.1) |
| M5 | 30 (32.6) | 17 (11.0) | 23 (11.6) |
| M6 | 3 (3.3) | 2 (1.3) | 0 |
| M7 | 0 | 3 (1.9) | 1 (0.5) |
| Unclassified | 12 (13.1) | 1 (0.6) | 131 (65.8) |
| Cytogenetic abnormality, n (%) | | | |
| No | 25 (27.2) | 79 (51.0) | |
| Yes | 28 (30.4) | 62 (40.0) | 46 (23.1) |
| Unknow | 39 (42.4) | 14 (9.0) | 153 (76.9) |
| allo-HSCT, n (%) | | | |
| No | 57 (62.0) | 94 (60.6) | 124 (62.3) |
| yes | 35 (38.0) | 61 (39.4) | 75 (37.7) |
| Follow-up, median（range）,years | 1.940 (0.003-8.478) | 1.000 (0.080-7.840) | 0.800 (0.005-4.055) |
| Status | | | |
| Alive | 36 (39.1) | 60 (38.7) | 117 (58.8) |
| Dead | 56 (60.9) | 95 (61.3) | 82 (41.2) |

SD, standard deviation; WBC, white blood cell; allo-HSCT allogeneic hematopoietic stem cell transplantation.

## Table S2. The primers for qRT-PCR.

| Target | Sequence 5' - 3' |
|---|---|
| β-actin (F) | TTGTTACAGGAAGTCCCTTGCC |
| β-actin (R) | ATGCTATCACCTCCCCTGTGTG |
| BATF (F) | TCGTATTGCCGCCCAGAAG |
| BATF (R) | ATCTCCTTGCGTAGAGCCG |
| EGR1 (F) | ACCCCTCTGTCTACTATTAAGGC |
| EGR1 (R) | TGGGACTGGTAGCTGGTATTG |
| PD1 (F) | CCAGGATGGTTCTTAGACTCCC |
| PD1 (R) | TTTAGCACGAAGCTCTCCGAT |
| PDL1 (F) | TGGCATTTGCTGAACGCATTT |
| PDL1 (R) | TGCAGCCAGGTCTAATTGTTTT |
| TIM3 (F) | GGAATACAGAGCGGAGGTCG |
| TIM3 (R) | CACCACGTTGCCACATTCAA |

**Table S3. Univariate and multivariate Cox regression analysis of AML patients in the JUN-dataset.**

| Variables | Univariate Cox regression | | Multivariate Cox regression | |
|---|---|---|---|---|
| | HR (95% CI) | *p* value | HR (95% CI) | *p* value |
| Age（ref: < 60） | | | | |
| ≥ 60 | 2.451 (1.410, 4.261) | 0.001 | 1.845 (1.008, 3.377) | 0.047 |
| Gender（ref: male） | | | | |
| female | 0.705 (0.412, 1.208) | 0.204 | | |
| WBC（ref: low） | | | | |
| high | 2.432 (1.428, 4.144) | 0.001 | 3.499 (1.969, 6.218) | <0.001 |
| HSCT（ref: no） | | | | |
| yes | 0.435 (0.242, 0.783) | 0.005 | 0.448 (0.230, 0.873) | 0.018 |
| BATF（ref: low） | | | | |
| high | 1.863 (1.052, 3.298) | 0.033 | 2.656 (1.460, 4.832) | 0.001 |
| EGR1（ref: high） | | | | |
| low | 1.741 (1.017, 2.980) | 0.043 | 2.092 (1.198, 3.655) | 0.009 |

**Table S4. Univariate and multivariate Cox regression analysis of AML patients in the TCGA and Beat-AML datasets.**

| Variables | Univariate Cox regression | | Multivariate Cox regression | | Univariate Cox regression | | Multivariate Cox regression | |
|---|---|---|---|---|---|---|---|---|
| | TCGA | | | | Beat-AML | | | |
| | HR (95% CI) | p value | HR (95% CI) | p value | HR (95% CI) | p value | HR (95% CI) | p value |
| Age（ref: < 60） | | | | | | | | |
| ≥ 60 | 2.883 (1.900, 4.376) | < 0.001 | 2.668 (1.718, 4.144) | <0.001 | 2.304 (1.478, 3.594) | <0.001 | 1.797 (1.134, 2.849) | 0.013 |
| Gender（ref: male） | | | | | | | | |
| female | 1.024 (0.684, 1.534) | 0.908 | | | 0.785 (0.506, 1.220) | 0.282 | | |
| WBC（ref: low） | | | | | | | | |
| high | 1.651 (0.997, 2.734) | 0.047 | 1.684 (1.010, 2.808) | 0.046 | 2.501 (1.307, 4.787) | 0.006 | 2.669 (1.353, 5.268) | 0.005 |
| HSCT（ref: no） | | | | | | | | |
| yes | 0.558 (0.364, 0.856) | 0.008 | 0.690 (0.435, 1.094) | 0.114 | 0.373 (0.229, 0.607) | <0.001 | 0.333 (0.201, 0.553) | <0.001 |
| BATF（ref: low） | | | | | | | | |
| high | 2.420 (1.589, 3.684) | < 0.001 | 2.287 (1.474, 3.550) | <0.001 | 3.018(1.771, 5.144) | <0.001 | 3.282 (1.855, 5.810) | <0.001 |
| EGR1（ref: high） | | | | | | | | |
| low | 1.984(1.227, 3.208) | 0.005 | 2.227 (1.357, 3.657) | 0.002 | 2.441 (1.565, 3.809) | <0.001 | 2.506 (1.585, 3.961) | <0.001 |

**Table S5. Univariate and multivariate Cox regression analysis of *BATF* and *EGR1* co-expression in the TCGA and Beat-AML datasets.**

| Variables | Univariate Cox regression | | Multivariate Cox regression | | Univariate Cox regression | | Multivariate Cox regression | |
|---|---|---|---|---|---|---|---|---|
| | TCGA | | | | Beat-AML | | | |
| | HR (95% CI) | *p* value | HR (95% CI) | *p* value | HR (95% CI) | *p* value | HR (95% CI) | *p* value |
| Age（ref: < 60） | | | | | | | | |
| ≥ 60 | 2.883 (1.900, 4.376) | <0.001 | 2.662 (1.716, 4.125) | <0.001 | 2.304 (1.478, 3.594) | <0.001 | 1.849 (1.168, 2.926) | 0.009 |
| Gender（ref: male） | | | | | | | | |
| female | 1.024 (0.684, 1.534) | 0.908 | | | 0.785 (0.506, 1.220) | 0.282 | | |
| WBC（ref: low） | | | | | | | | |
| high | 1.651 (0.997, 2.734) | 0.047 | 1.723 (1.024, 2.899) | 0.040 | 2.501 (1.307, 4.787) | 0.006 | 2.473 (1.265, 4.834) | 0.008 |
| HSCT（ref: no） | | | | | | | | |
| yes | 0.558 (0.364, 0.856) | 0.008 | 0.693 (0.445, 1.080) | 0.105 | 0.373 (0.229, 0.607) | <0.001 | 0.345 (0.208, 0.572) | <0.001 |
| BATF/ EGR1（ref: BATFlo/ EGR1hi） | | | | | | | | |
| BATFhi / EGR1lo | 6.246 (3.186, 12.244) | <0.001 | 5.365 (2.720, 10.583) | <0.001 | 6.045 (2.877, 12.704) | <0.001 | 5.638 (2.607, 12.191) | <0.001 |
| BATFhi /EGR1hi or BATFlo/EGR1lo | 2.175 (1.371, 3.450) | 0.001 | 2.075 (1.295, 3.326) | 0.002 | 2.462 (1.542, 3.932) | <0.001 | 2.729 (1.679, 4.437) | <0.001 |
